# Models of the interaction of metal tips with insulating surfaces

**DOI:** 10.3762/bjnano.3.37

**Published:** 2012-04-13

**Authors:** Thomas Trevethan, Matthew Watkins, Alexander L Shluger

**Affiliations:** 1 Department of Physics and Astronomy, University College London, Gower Street, WC1E 6BT London, United Kingdom; 2 WPI-AIMR, Tohoku University, 2-1-1 Katahira, Aoba, Sendai, 980-8577, Japan; 3 Department of Chemistry, University of Sussex, Brighton, BN1 9RH, United Kingdom; 4 The London Centre for Nanotechnology, University College London, 17–19 Gordon Street, WC1H 0AH London, United Kingdom

**Keywords:** atomic force microscopy, density functional theory, ionic surfaces, metallic asperities, surface interactions

## Abstract

We present the results of atomistic simulations of metallic atomic-force-microscopy tips interacting with ionic substrates, with atomic resolution. Chromium and tungsten tips are used to image the NaCl(001) and MgO(001) surfaces. The interaction of the tips with the surface is simulated by using density-functional-theory calculations employing a mixed Gaussian and plane-wave basis and cluster-tip models. In each case, the apex of the metal cluster interacts more attractively with anions in the surfaces than with cations, over the range of typical imaging distances, which leads to these sites being imaged as raised features (bright) in constant-frequency-shift images. We compare the results of the interaction of a chromium tip with the NaCl surface, with calculations employing exclusively plane-wave basis sets and a fully periodic tip model, and demonstrate that the electronic structure of the tip model employed can have a significant quantitative effect on calculated forces when the tip and surface are clearly separated.

## Introduction

The noncontact atomic force microscope (NC-AFM) is capable of imaging both conducting and insulating systems with true atomic resolution and has provided extraordinary contributions to surface science [1–3]. In NC-AFM the tip is prevented from jumping into mechanical contact with the sample surface due to the large restoring force of the cantilever at the turning point of the tip trajectory when it is closest to the surface. As a result, the instrument can probe all regions of the tip–surface interaction with high stability, in particular the “near contact” region of separation where the tip apex atom and surface are separated by only a few angstroms (i.e., the typical range of chemical bonds). However, the nature of the force between the tip and the surface is highly dependent on the exact atomic structure and chemical nature of the tip apex. In the case of ionic surfaces, different terminating atoms can lead to completely inverted image contrasts [3–4], in which case it is not even possible to identify the polarity of surface ions corresponding to protrusions in the image. The control and characterization of the tip-apex termination is therefore critical for the reliable interpretation of images.

AFM tip–cantilever assemblies are usually fabricated from silicon, which is then exposed to air and will thus develop a native oxide layer with air-induced contaminants. This layer can be removed in situ inside the ultrahigh vacuum chamber, by sputtering and/or annealing. However there is no guarantee that the tip apex is pure silicon, and contaminant atoms or molecules may remain. The tip can also be contaminated by material from the surface during imaging; in fact, in many cases atomic-resolution images are only obtained after the tip has been deliberately crashed into the surface, implying that the tip apex is formed from surface species [1–2]. The development of NC-AFM based on a quartz tuning fork (qPlus sensor) instead of a silicon cantilever has led to more freedom in choosing the tip material, as a tip can be manually attached to the tuning-fork prong [5]. However, the problem of keeping the tip apex free of contaminants remains.

One approach to deal with the problem of tip–apex control is to employ a tip material that is easy to prepare and characterize in situ, i.e., in UHV and through the tip–surface interaction. The use of metal-coated tips meets both of these requirements. Firstly, coating a standard silicon tip with a layer of metal can be achieved in the UHV chamber by evaporation (assuming that the metal bonds effectively to the oxide layer) [6], resulting in a high confidence that the metallic tip apex is free from airborne contaminants. Secondly, it is possible to judge based on the conductivity of the tip as to whether the tip apex is metallic or terminated with contaminant atoms. This can be achieved by recording the resonant frequency while the bias voltage, applied between the tip and the sample holder, is varied. As described in [7], smooth parabolic curves that are independent of the scan direction indicate a metallic tip apex. On the other hand, discontinuities and hysteresis between scan directions indicate charge localization and reconfiguration and a tip apex that is not truly metallic.

It has been demonstrated that a chromium-coated tip is capable of imaging the bulk NaCl(001) surface with atomic resolution, at relatively large tip–surface separations (i.e., >5 Å), reducing the potential for the tip to become contaminated by the surface [7]. Plane-wave density-functional-theory (DFT) calculations employing a periodic metallic-tip model demonstrated that the Cr tip apex interacts most strongly with anions (Cl^−^) in the surface, and that these ions correspond to protrusions in the image. Thus these experiments and the accompanying calculations suggest that properly characterized Cr coated tips can be used to unambiguously interpret the contrast in images of the NaCl(001) surface. The mechanism of contrast formation proposed is quite universal and involves the interaction of the polarized tip (due to the Smoluchowski effect) with the surface ions at large tip–surface separations and the hybridization of tip and anion states at smaller separations. Therefore it is reasonable to expect that similar mechanisms should apply both to other ionic surfaces and to other metals. A more general understanding of the interaction of metallic tips with ionic surfaces will help motivate experimental efforts and inform choices of tip material and tip-preparation methods.

In this paper we present the results of atomistic DFT calculations performed to investigate the interaction between metal tips and the typical binary ionic surfaces, NaCl(001) and MgO(001). The high symmetry of these surfaces makes their AFM images particularly difficult to interpret [3], although in the case of NaCl(001), there have been several approaches to successfully interpret atomic-resolution images [8–11]. We consider two types of metal tip, namely chromium and tungsten, which are chosen due to their common use in scanning-probe experiments. For several different combinations of tip and surface, we determine the tip–surface force field and the origin of the tip–surface interaction at close approach. These calculations employ cluster-tip models and localized Gaussian atomic basis sets, which result in a significantly lower computational cost when compared with fully periodic tips (which consist of a significantly greater number of atoms) and plane-wave calculations. We compare the results of these two approaches for the Cr/NaCl system and discuss the effect of the DFT methodology and the electronic structure of the tip model on the accuracy of the calculations of tip–surface forces. The plan of the rest of the paper is as follows: The next section describes the methodology employed; the third section describes the results of the calculations; and in the final section a discussion of the results and how they compare to other calculations is presented.

## Results and Discussion

The calculations presented in this study were performed by using the DFT module of the CP2K code [12] and employing the PBE exchange-correlation functional [13]. Gaussian basis sets of DZVP quality were used with semicore GTH pseudopotentials [14–16]. The pseudopotentials included 9, 10, 14 and 18 valence electrons for Na, Mg, W and Pt. The auxiliary plane-wave basis, used to calculate the Hartree energy, had an energy cutoff of 4000 eV. To account for the metallic nature of the tip (i.e., a very small band gap) in the simulation, we also employ Fermi–Dirac smearing of the molecular-orbital occupation numbers, with an electronic temperature of 2500 K.

Both the NaCl(001) and MgO(001) surfaces were modeled using a periodic slab, 6 × 6 atoms in area and three atomic layers deep, where the bottom-most layer is frozen in bulk-like positions. For a direct comparison with the results of previous plane-wave calculations employing a periodic-tip model, the NaCl(001) surface was also modeled with a 5 × 5 primitive unit cell surface area, three atomic layers deep, which was chosen to match the *x*-*y* periodicity of the periodic tip model. The slabs are periodic in the *x*-*y* directions, and there is a vacuum gap of 30 Å in the *z*-direction. The lattice separation in the NaCl slab is 2.78 Å and in the MgO slab is 2.12 Å. When the geometries of the surface slabs are optimized they exhibit rumpling, with the anions protruding from the surface plane. The corrugation of the NaCl surface is approximately 0.1 Å and 0.04 Å in MgO. The one-electron band gaps for the NaCl surface at 4.9 eV, and for the MgO surface at 3.6 eV, are underestimated, which is typical for PBE calculations.

**Figure 1:**
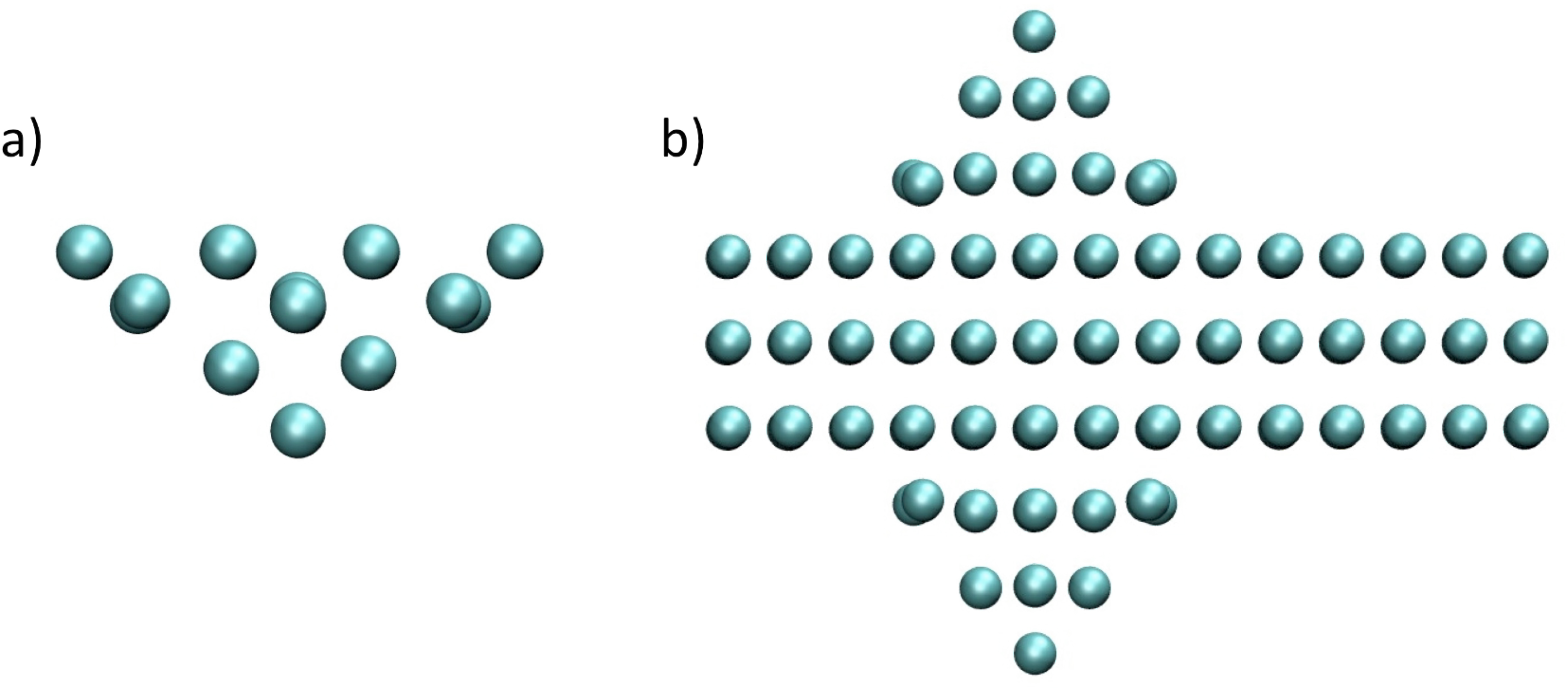
(a) Side-on view of the structure of the Cr and W cluster tip models. (b) The structure of the periodic Cr tip model.

The tip models are shown in Figure 1. The cluster Cr and W tips consist of four-layered pyramids, cut from the body-centered-cubic (BCC) structure of the bulk crystals. The top two layers of the 30 atom tips are frozen, and the lower two layers are free to relax. For a direct comparison with the plane-wave calculations presented in [7], a periodic-tip model consisting of a three-layer BCC slab of Cr with symmetric pyramidal protrusions (Figure 1b) was also employed. It is well-known that the structure and morphology of the tip has a significant effect on the tip–surface interaction [17–18]; however, this type of pyramidal protrusion was shown to be the best match to the experimental measurements reported for this system [7]. The work functions for the Cr tips are calculated as being approximately 3.7 eV for both tip models, which is similar to previous calculations for the Cr surface [19] but slightly less (by 0.2–0.6 eV) than the experimental values [20–21]. For all of the tip models the Fermi energy lies well within the band gap of the ionic surface slabs.

To calculate the tip–surface force field, the frozen part of the tip is fixed at a position above the surface, the system relaxed, and the total energy calculated. The tip is then moved a small distance closer to the surface, and this is repeated to map out the energy as a function of the tip position. The gradient of this energy in the *z*-direction is then used to determine the tip force. The tip height is defined as the separation that would exist between the front atom of the tip and the surface plane if there were no relaxation in the tip (i.e., with the tip away from the surface). The DFT method is known to underestimate atomistic dispersion forces; however, these are not expected to contribute to the atomic-scale variation of the force on the tip above different atomic sites [3]. A macroscopic van der Waals attraction is added to the total force on the tip for simulated image calculations, as stated in the Experimental section.

To correct for the basis-set-superposition error (BSSE), which acts to increase the force on the tip originating from the interaction with the surface, due to the overlap of the basis functions of the surface and tip, we employ the counterpoise method to correct the total system energy for different tip positions relative to the surface [22]. Our calculations demonstrate that the BSSE is similar at a given tip height above both anions and cations (approximately 0.1 eV at 4 Å), and is therefore not likely to contribute to atomic-scale contrast. Furthermore, the BSSE is only present at tip–surface separations below 4.5 Å, as above this height there is no orbital overlap.

**Figure 2:**
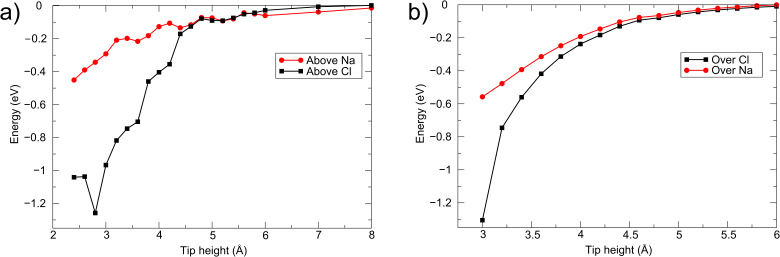
(a) Energy as a function of cluster Cr tip height above the NaCl(001) surface. (b) Energy as a function of tip height above the MgO(001) surface.

The total energy as a function of tip height, for the apex of the Cr cluster tip directly above both Cl^−^ and Na^+^ ions in the NaCl surface, and above both O^2−^ and Mg^2+^ ions in the MgO surface, is shown in Figure 2. Here the energy change is relative to the energy of the tip and surface when they are completely separated. In each case it is clear that the force is largest directly above anions in the surface, significantly so in the range probed by noncontact imaging, i.e., 3–5 Å. For each tip above an anion in the surface, at close approach (approx. 3–4 Å) the force increases markedly due to a structural change consisting of strong displacement of an anion out of the surface to bond to the tip apex. This jump of a surface ion to the tip apex will result in hysteresis in the tip–surface force field and atomic-scale dissipation being measured by the NC-AFM instrument [23–24]. For the Cr tip interacting with the NaCl surface, the total charge on the tip at a separation of 6 Å is less than −0.01 |*e*| (from a Mulliken population analysis); however, when the tip comes closer to the surface above a Cl^−^ ion, there is a small charge transfer to the tip (of −0.03 |*e*| at a height of 4 Å and of −0.1 |*e*| at 3 Å). For the tip above the MgO surface, a similar transfer occurs, but it is slightly more pronounced (a charge on the tip of −0.16 |*e*| at 4 Å above an O^2−^ ion in the surface).

**Figure 3:**
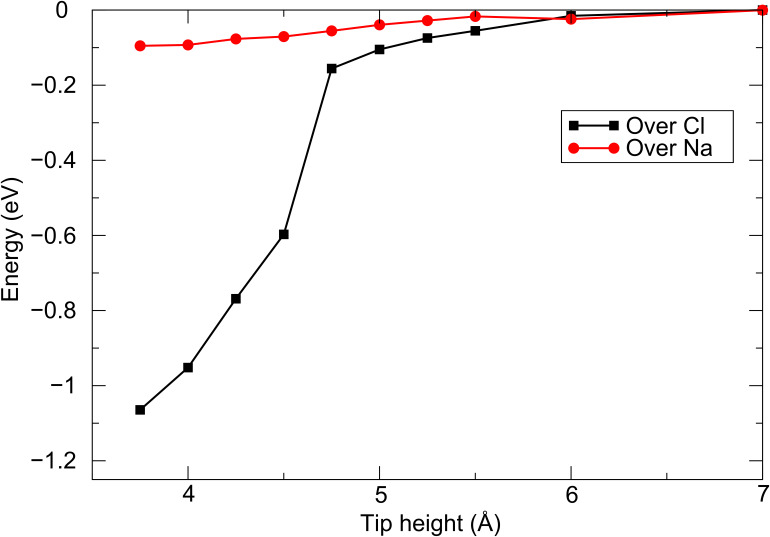
Energy as a function of tip height for the W tip interacting with the NaCl(001) surface.

Figure 3 shows the total energy as a function of the tip height for the W tip directly above Cl^−^ and Na^+^ ions in the NaCl(001) surface. As before, the interaction is strongest above the anion, and increases significantly below 4.5 Å (note this is not due to an instability caused by an atom jump). The charge transfer to the tip at close approach is similar to that in the case of the Cr tip interacting with this surface, which is to be expected due to the similar Fermi energies of the two clusters. In the case of both tips, the origin of the charge transfer at close approach and the increased tip force above the anions is due to the hybridization of the d states in the tip apex atom with the p states in the surface anion.

**Figure 4:**
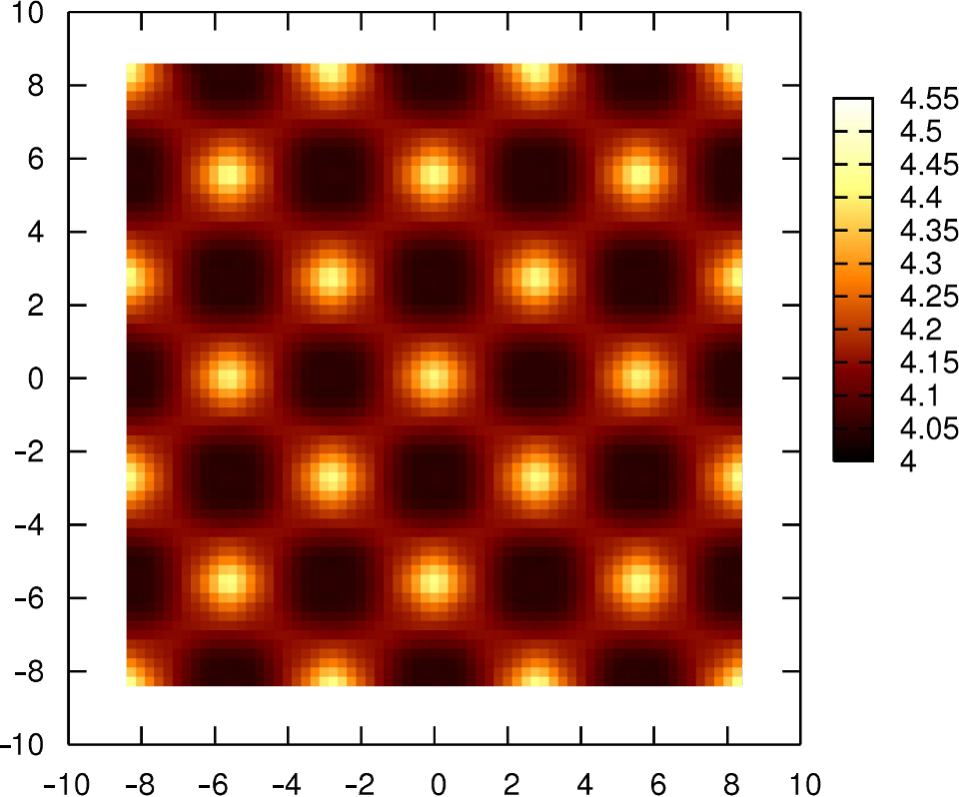
Constant-frequency-shift image (Δ*f* = −60 Hz) of the NaCl surface imaged with the cluster Cr tip.

In each of the tip–surface combinations, the calculated force fields would result in the anions being imaged as prominent protrusions in a constant-frequency-shift image of the surfaces. To demonstrate this, and show the extent of typical atomic scale corrugation, we simulated the imaging of the NaCl surface with the Cr cluster tip, using typical imaging parameters based on a traditional silicon cantilever (listed in the Experimental section). The force field used for these calculations was calculated on a lateral square grid with a spacing of 1/8 of the lattice constant between points (i.e., four points between adjacent surface ions), and between tip heights of 3 Å and 7 Å. Figure 4 shows a constant-Δ*f* image (Δ*f* = −60 Hz) of the NaCl surface, in which the distance of closest approach is 3.6 Å. The rumpling is approximately 0.6 Å with the protrusions corresponding to Cl^−^ ion lattice positions and depressions to Na^+^ ion positions.

To investigate both the contribution of the electronic structure of the tip and the type of simulation method to the interaction between a metallic tip and an ionic surface, we calculated the changes in total energy as a function of tip position for the periodic Cr tip model interacting with the NaCl surface. We used the exact same system configurations as used in previous plane-wave DFT calculations, employing the VASP code [25] (as described above). The same PBE correlation-exchange functional employed in [7] was used here. The main difference in the model we apply is in the form of the basis functions, in which the wave function of the system is expanded: Here they are Gaussian and atom-based, as opposed to being plane waves.

**Figure 5:**
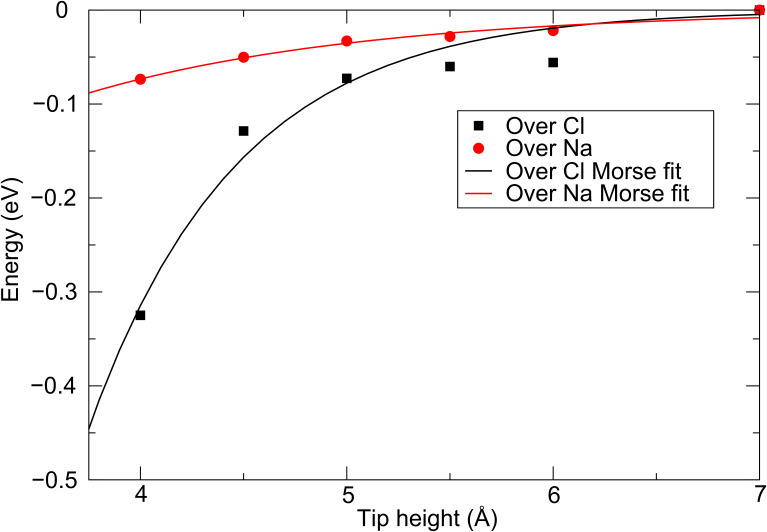
Total energy changes as a function of tip height for the periodic Cr tip interacting with the NaCl(001) surface, and Morse function curves fitted to the data points.

**Figure 6:**
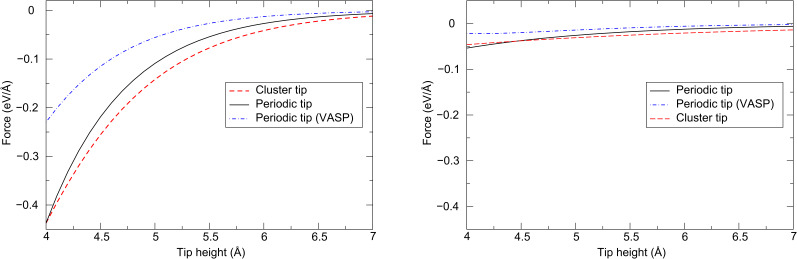
Tip force as a function of height directly above Cl^−^ (left) and Na^+^ (right) ions in the NaCl(001) surface, for the cluster tip and periodic tip, and an identical periodic tip but with energies determined from plane-wave (VASP) calculations [7].

Figure 5 shows the total energies (BSSE corrected) as a function of tip position (the exact same positions calculated in [7]). As in [7], Morse bond functions were fitted to these energies as a function of tip height for each position, in the noncontact range of 4–7 Å, where no instabilities occur. The derivative of this function gives the force on the tip due to the interaction with the surface, as a function of tip height, which is shown in Figure 6 in the range of 4–6 Å, along with the curves from the plane-wave calculations presented in [7], and fitted curves for the cluster Cr tip model discussed above. These forces show that the periodic tip model leads to an overall force that is quantitatively smaller than that in the cluster model for a given tip height, by approximately 10% in the 4.5–5.5 Å range.

The absolute forces between the NaCl surface and the periodic tip model above both Cl^−^ and Na^+^ ions, as calculated in this study, are larger than the forces calculated by using exactly the same setup in the previous plane-wave calculations: The forces are larger by approximately 50–100% in the 4.5–5.5 Å range.

## Conclusion

We have presented the results of calculations to determine how metal-cluster tips interact with two representative, model ionic surfaces at typical NC-AFM imaging distances. These calculations all unambiguously show that the attractive force on the tip will be strongest with the tip apex positioned directly above an anion in the surface, over the entire range of NC-AFM imaging distances (3–6 Å). As a result, the anion will always be imaged as elevated (bright) in NC-AFM images of these surfaces with these tip materials. The origin of the tip–sample interaction close to the surface is due to hybridization of the anion p states with the d states of the tip apex. This interaction mechanism does not give rise to contrast further from the surface (i.e., >4.5 Å); however, the force is still significantly greater above the anion beyond this distance. As was determined in [7], the interaction of the tip with the surface beyond this distance is purely electrostatic: In a truly metallic tip, the tip apex develops a small intrinsic dipole due to the Smoluchowski effect. The positive end of this dipole points to the surface and increases the interaction over the anions. In addition, anions move out from the surface due to the surface rumpling and are also, in general, more easily polarized than are cations. Both of these effects enhance the attractive tip–surface force above the anions. Here, the induction energy is −1/2 α|*E*|, where α is the atomic polarizability of the tip apex atoms and *E* is the electric field generated by the interaction, which is reproduced implicitly in the DFT calculations.

Each of the tip models employed in these calculations (cluster tip, periodic tip) give similar qualitative results, in so much that the force is strongest over the anion. This supports our previous conclusion that using well-characterized metallic tips may enable unambiguous chemical identification of image features [7]. It is not particularly surprising that quantitative differences between forces are obtained upon using different tip models and computational methods, as we push the accuracy of the calculations at large tip–surface separations. In particular, the cluster model leads to a slightly larger overall attractive force in the 4–6 Å distance range than does the periodic model, which may be due to an increased reactivity due its small size. For the periodic tip model, the tip–surface forces calculated in this study are also quantitatively different to in the calculations presented in [7], in which a plane-wave basis set was employed but with the same functional, even though again they qualitatively agree. Overall, the total attractive force at a given separation (in the near-contact range) is up to 100% larger, even when exactly the same atomic configuration is employed; although in absolute terms the difference in the forces is small. In this noncontact distance range, the asymptotic behavior of the electronic density (which may be significantly affected by the basis functions employed), the different treatment of the long-range electrostatics and periodic boundary conditions, and/or slight differences in the effective polarizabilities of the surface or tip ions may all contribute to the observed force difference. The polarizability could be affected by the quality of the basis set, *k*-point sampling and the pseudopotential used (the plane-wave code uses a pseudopotential constructed with Cr in a d^5^s^1^ state, whilst the present calculations include s^2^p^6^d^5^s^1^). At present, the full convergence of all the parameters in these calculations is at the limits of the available computational resources, and detailed investigations to disentangle the subtle differences between the calculations are not feasible. These results demonstrate that when calculating weak forces between a metallic tip and surface for a quantitative comparison with experimental results, care must be taken over the choice of both the tip model and the calculation method: Both the electronic structure of the tip and the method can have a significant effect on the calculated forces.

## Experimental

### Simulated image parameters

Elastic constant: 148.7 N/m; natural frequency: 189000.0 Hz; setpoint amplitude: 5 nm; Q-factor: 10000.0. Macroscopic van der Waals: Hamaker constant: 0.999 eV; Tip radius: 18.0 nm
